# Demographics of Youth With Newly Diagnosed Acute/Recent HIV Infection in Adolescent Trials Network 147: Early Treatment of Acute HIV Infection

**DOI:** 10.1016/j.jadohealth.2023.09.017

**Published:** 2023-12-03

**Authors:** Tara Kerin, Ruth Cortado, Sophia G. Paiola, Justine Ceballos, Sue Ellen Abdalian, Risa Flynn, Robert Bolan, Yetunde V. Adebambo, Myung Shin Sim, Dallas Swendeman, Manuel A. Ocasio, Jasmine Fournier, Bonnie Ank, Yvonne Bryson, Karin Nielsen-Saines

**Affiliations:** a Department of Pediatrics, David Geffen School of Medicine, University of California Los Angeles, Los Angeles, California; b Department of Pediatrics, School of Medicine, Tulane University, New Orleans, Louisiana; c The Los Angeles LGBT Center, Los Angeles, California; d Department of Psychiatry and Behavioral Sciences, University of California Los Angeles, Los Angeles, California; e UCLA Department of Medicine Statistics Core, University of California Los Angeles, Los Angeles, California

**Keywords:** HIV, Acute HIV infection, youth HIV

## Abstract

**Purpose:**

Gay, bisexual, and other cisgender men who have sex with men, and racial minority youth are at elevated risk of acquiring HIV infection. The Adolescent Trials Network 147 recruited youth with acute/recent HIV-infection for early antiretroviral treatment. The cohort make-up is described here.

**Methods:**

Treatment-naïve, recently identified HIV + youth, aged 12–24 years, from Los Angeles and New Orleans were recruited from community centers, clinics, social media, and a high-risk seronegative cohort (n = 1,727, the Adolescent Trials Network 149) using point-of-care assays. Acute HIV infection was determined by Fiebig staging. HIV RNA viral load (VL) and CD4 cell counts, along with demographic and behavioral data were assessed at enrollment.

**Results:**

Between July 2017 and July 2021, 103 newly diagnosed youth were enrolled, initiating antiretroviral treatment within a week. Mean age was 20.8 years (standard deviation: 2.4); 90.3% identified as cis male, 83.5% were single or in casual relationships, 71.8% were gay, bisexual, and other cisgender men who have sex with men; 60.2% were Black. One-fourth (24.3%) reported homelessness ever; 10.7% within last 4 months. At enrollment, median plasma VL was 37,313 HIV RNA copies/ml (interquartile range: 5,849–126,162) and median CD4 count 445.5 cells/mm3 (interquartile range: 357–613). 40% of youth reported acute retroviral symptoms before or at enrollment. Acutely infected, seroconverting youth had the highest VL. Sexually transmitted coinfections were present at enrollment in 56% of the cohort, with syphilis being most frequent (39%).

**Discussion:**

Early identification and treatment of HIV can increase positive HIV outcomes. A high sexually transmitted infection burden was present in recently HIV-infected youth. Acute retroviral symptoms were not reported by most participants, demonstrating that broad universal HIV screening is needed for identification of recent infection in youth.

Adolescents who are displaced and living in shelters or in the streets constitute an extremely vulnerable population for acquisition of HIV infection worldwide. In the United States, homeless youth, particularly minorities and LGBTQ+ youth are very susceptible to substance abuse, juvenile justice contact, and acquisition of HIV and other sexually transmitted infections (STIs). The displaced adolescent population is not generally amenable to routine clinic follow-up and potentially could be more easily identified through mobile outreach efforts. HIV prevalence in this group can be as high as 5.3%, as indicated in an early study [1], while recent estimates fall between 2% and 4.5% in this population [2]. Although HIV incidence is unknown, high rates of concurrent exposures to other STIs, substance abuse, and survival sex suggest acute infection is high in this population.

As part of the Adolescent Trials Network (ATN) Comprehensive Adolescent Recruitment and Engagement Strategies initiative, we conducted a study of Acute HIV Infection in Youth (ATN 147). This study was developed to assess potential differences between adolescents with acute HIV infection (within 90 days of infection) or those with recent infection (>90 days of infection) as measured by Fiebig staging [3]. Pediatric studies of HIV perinatally infected infants treated very early with potent antiretroviral therapy [4–7] as well as studies of adult cohorts treated during acute infection [8–12] demonstrate that very early treatment of HIV is associated with plasma viral suppression and decrease in viral reservoir burden [13–15]. Preservation of the immune system through early detection and treatment is likely predictive of long-term HIV control. In this paper, we examine the baseline differences of the acute versus the nonacute infected youth.

At the time ATN 147 was initiated in Los Angeles and New Orleans (July 2017), most HIV-infected adolescents between the ages of 13–24 living in the United States (youth living with HIV: YLHIV) were infected via sexual transmission. Among adolescent females, 48% acquired infection via heterosexual contact, 42% perinatally, and 5% via injection drug use alone [16]. Among diagnosed adolescent males (who constitute the vast majority of adolescents with HIV), 82% acquired infection via male-to-male sexual contact, 10% perinatally, 3% via heterosexual contact, 3% via male-to-male contact and injection drug use, and 1% via injection drug use alone [16]. Consequently, gay and bisexual adolescent males comprise the adolescent population at greatest risk of HIV infection [17]. In the present analysis we report demographics, social history, clinical characteristics, estimated duration of HIV infection (acute vs. nonacute) and coinfections at baseline for the population of youth enrolled in ATN 147.

## Methods

### Design

Adolescent Trials Network (ATN) 147 is a longitudinal strategic prospective treatment study that identifies, promptly treats, and follows a cohort of adolescent or young adults aged 12–24 years with acute (Fiebig stage I–V, infection within the past 90 days), and recent/established HIV (Fiebig Stage VI, more than 90 days since infection). All eligible study participants are treatment naive and recently diagnosed with HIV. The study measures the effects of early antiretroviral therapy on the achievement and durability of HIV plasma virus suppression, establishment and persistence of HIV-1 reservoirs and HIV antibody responses. Youth are treated according to the United States Department of Health and Human Services standard of care and followed for a period of 24 months. This analysis focuses on the demographics of the cohort at baseline, comparing differences between acutely infected and nonacutely infected participants.

### Population

Treatment naïve youth, aged 12–24 years of age in Los Angeles and New Orleans, were recruited from direct referrals or through the complementary U19-funded study of high-risk HIV-seronegative youth: Cost-Efficient Interventions for Youth at Risk for HIV (ATN 149) [18].

### Study sites and recruitment

Participating ATN 147 study sites include David Geffen University of California at Los Angeles School of Medicine, Department of Pediatrics, Division of Infectious Diseases, the Los Angeles LGBT Center, and Tulane University School of Medicine, Department of Adolescent Medicine. The study had two distinct methods of enrollment. One, potential participants in ATN 149 were screened at recruitment sites in Los Angeles and New Orleans for HIV high-risk behavior and tested for HIV using point of care testing including the Alere HIV-1/2 rapid test (Waltham, MA) every 4 months, which indicates the presence of HIV-1 antibody and/or antigen [19]. HIV-positive youth were then referred to enroll in ATN 147 upon evidence of acquisition of HIV infection [3,19]. Second, ATN 147 also enrolled any newly HIV diagnosed, treatment-naive youth referred for care to University of California at Los Angeles and LGBT in Los Angeles and Tulane University in New Orleans [3].

### Study inclusion and exclusion criteria

Inclusion criteria included: (1) age of 12–24 years, (2) a positive HIV result (Alere rapid test and GeneXpert HIV qualitative polymerase chain reaction [PCR]), (3) ability and willingness to provide written informed consent, and (4) willingness to initiate ART. Exclusion criteria included (1) prior ART use (>1 week); (2) active drug or alcohol use or dependence that would interfere with adherence to study requirements; (3) any acute, chronic, or recent and clinically significant medical condition that would interfere with adherence to study requirements or jeopardize the safety or rights of the participant; (4) chronic or recurrent use of medications that modify host immune response, for example, oral or parenteral steroids and cancer chemotherapy; (5) clinical treatment with an ART regimen less effective than those recommended by DHHS HIV clinical guidelines and; (6) enrollment in an experimental ART regimen. Other eligible youth were referred from urgent care sites, emergency departments, and other clinics.

### Laboratory evaluations and assessments

#### Fiebig staging.

Acute HIV infection was determined using the Clinical Laboratory Improvement Amendments-waived Alere (Waltham, MA), an HIV-1 p24 antibody and antigen test, as well as with the Cepheid (Sunnyvale, CA) Xpert HIV-1 Qual Assay to detect HIV-1 total nucleic acids, and HIV-1 Western blot (BioRad, Hercules, CA). Based on the results of these tests, a Fiebig stage [20] was assigned to each participant. Fiebig stages 1–5 were classified as acute, while Fiebig stage 6 (as defined by the presence of the p31 band on Western blot) was classified as recent/established infection at baseline.

#### Laboratory testing at study entry.

Baseline testing has been previously described [3,18,19,21], but briefly, chlamydia and gonorrhea were tested using a Food and Drug Administration-approved Cepheid Xpert chlamydia/gonorrhea Assay PCR (Sunnyvale, CA) on throat and rectal specimens. Syphilis was tested using the Clinical Laboratory Improvement Amendments-waived Syphilis Health Check finger stick blood test to detect treponemal antibodies, and positive results were followed with rapid plasma regain in serum. A positive result in any of the tests was classified as a positive STI result. Hepatitis panel was performed with hepatitis A, B, and C serologies and tuberculosis-Gold quanti-FERON (Quiagen, Germantown, MD) testing. Clinical laboratories included a complete blood count with differential and platelets and a chemistry panel, including liver and kidney function tests. Entry clinical forms noted self-reported symptoms at enrollment, before enrollment, or no symptoms of acute retroviral infection noticed.

#### HIV RNA PCR and CD4/CD8 T cell subsets.

Quantitative plasma HIV RNA limit of detection < 40cp/ml (Abbot) and CD4/CD8 T cell counts were collected at baseline, before ART initiation. ART was started within a week of initial baseline visit for all patients.

#### Antiretroviral treatment.

At the ATN 147 enrollment visit, following provision of written informed consent, youth were prescribed ART, and clinically treated according to standard of care HIV-1 management as defined in US DHHS guidelines [22]. Geno-typic drug resistance testing was performed at the baseline visit. Fixed-dose combination regimens were generally preferred favoring once-daily integrase inhibitor-based regimens (Gilead Sciences, Foster City, CA), specifically Genvoya, (elvitegravir, cobicistat, emtricitabine, tenofovir alafenamide), Biktarvy (bictegravir, emtricitabine, tenofovir alafenamide), and Odefsey (emtricitabine, rilpivirine, tenofovir alafenamide). Prompt access to antiretroviral therapy was made possible through assistance with enrollment into public HIV antiretroviral treatment programs.

#### Behavioral assessments.

Behavioral interviews were administered at baseline from a detailed questionnaire. Demographic details including race/ethnicity, gender, sexual orientation, education, and employment were collected along with additional behavioral and psychological assessments of stress, anxiety, drug and alcohol use, sexual partners, and sexual activities.

#### Study visits.

Enrollment visits occurred on day 0, and subsequent study visits occurred on day 7 postenrollment, weeks 2 and 4, and at 4, 8, 12, 18, and 24 months following enrollment. The present analysis is restricted to the enrollment period.

### Data and analysis

Clinical data were extracted from medical records and behavioral data were collected from in-person visits using the CommCare software (Dimagi Inc, Cambridge, MA) as previously described [23]. Categorical comparisons between acutely infected and nonacutely infected participants were performed using Chi-square analysis or fisher’s exact test when appropriate. Between-group comparisons of continuous variables was achieved using t-tests or Wilcoxon Rank-Sum test for nonparametric variables. Analysis was done using R [24].

#### Human subjects approval and ethical considerations.

Adolescent Trials Network (ATN) 147 was reviewed and approved by the institutional review board of all participating institutions. Study participants were paired with counselors and interviewers. Detailed assessments were made at each study visit of the patient’s mental status and outside activities, including the presence of other STIs and substance abuse. Participants were referred to mental health care, and resources for housing and employment were made available as needed. Study participants were screened for depression at each visit, and if needed were referred to a mental health provider for further assessment.

## Results

Study enrollment began in July 2017 and concluded in July 2021. An average of two participants a month were enrolled during the study over 4 years, with half of the participants enrolled in Los Angeles (N = 51) and the other half in New Orleans (N = 52) (Table 1). The cohort had an average age of 20.8 years, and the majority identified as cis male (90.3%), single or in one or more casual relationships (83.5%), same gender sexual orientation (71.8%) and Black or African-American (60.2%). More than half of the participants had some higher education past high school (52.4%) and most where either employed (49.5%) or attending school (29.1%). However, almost one fourth (24.3%) reported that they had once been homeless, and 10.7% were homeless within the last 4 months. Clinically, the participants presented a median plasma viral load (VL) of 37,313 HIV RNA cp/ml (interquartile range (IQR): 5,849–126,162), median CD4 count of 445.5 cells/cc (IQR: 357–613), 54% had an additional STI diagnosis at enrollment, and almost 40% reported acute HIV symptoms, such as fever, fatigue, headache, sore throat, muscle pain, lymphadenopathy, and skin rash, before (12.6%) or at (27.2%) enrollment. No participants reported AIDS defining conditions, but one participant had a CD4 count of less than 200, and seven others had absolute CD4 counts under 250. Tuberculosis was reported in one patient (data not shown) While some participants enrolled already having private healthcare (17.5%), most participants either had, or were linked to at the time of enrollment, to a government assisted healthcare plan (Table 1). Almost all patients were prescribed regimens which included nucleoside reverse transcriptase inhibitor (NRTI)/integrase strand transfer inhibitor combinations at baseline, apart from three participants prescribed a non-nucleoside reverse transcriptase inhibitor/NRTI combination. Over two thirds (68%) of participants reported starting ART within 24 hours of enrollment into the study, 10 more (9.7%) started within 48 hours of enrollment, and the remaining participants started ART within the first week. Although all participants were ART-naïve, 16 youth (15.5%) had pre-existing non-nucleoside reverse transcriptase inhibitor, NRTI, and/or major protease inhibitor resistance mutations at enrollment.

Participants were stratified by Fiebig Stage in Table 2 as either acutely infected (Fiebig Stage I-V, N = 36) or as recently infected, but more than 90 days before enrollment (Nonacute, Fiebig VI, N = 67). No significant difference in age, race/ethnicity, gender, sexual orientation, or insurance could be seen between acutely and recent/established HIV infection groups. There was a significant difference in median plasma VL between acutely infected participants (86,900 cp/ml, IQR: (9,741–559,230) and recent/established infections (32,334, IQR: (3,755–76,552), *p* = .006); median CD4 count (acute: 517 (IQR: 418–686), recent/established: 424, (IQR 344–570), *p* = .02), and more acutely infected participants reporting symptoms around enrollment (*p* = .008). Significant differences by study site were seen by recruitment source (*p* < .001), as the New Orleans site enrolled more participants from the high-risk seronegative ATN 149 study (77.4%). Stratification by recruitment source showed significant differences between age groups, with ATN 149 enrollees being younger (*p* = .03) and identifying more as Black or African-American (*p* = .04) than patients not initially enrolled in ATN 149. No differences were observed in STIs at baseline, gender or sexual orientation, or insurance (Table 2).

Table 3 shows behavioral variables stratified by participant Fiebig stage. No differences in employment or education were noted across groups. Additionally, no difference was seen between any groups regarding homelessness (recent or ever), sex in exchange for money or services (recent or ever) or having an HIV positive partner in the last 4 months. Almost a third of participants reported a suicide attempt in their lifetime (N = .30), and six reported an attempt in the last 4 months. There were no differences by recruitment or Fiebig stage for recent suicide attempts, but acutely infected participants reported more suicide attempts in their lifetime than those with recent/established infections (*p* = .03). Regarding mental health treatment, five participants had been hospitalized for mental health care in their lifetime, with two reporting a hospital stay in the last 4 months. Lifetime outpatient services for mental health (including therapy, counselor, social worker or psychiatrist) were reported by 25 participants, with 18 reporting services in the last 4 months (Table 3). Of the 25 participants who sought outpatient mental health services, only 5 (20%) had private insurance, although there was no significant difference in insurance type and outpatient services (data not shown).

Examining recent drug and alcohol use, marijuana was the most-reported drug taken in the last 4 months by all participants (N = 76, 74%). No significant difference in self-reported use in the last 4 months by Fiebig stage for any drug was evident (Table 3). Lifetime drug use illustrated heavier use of all drugs in all participants; however, only opiates (*p* = .04) and phencyclidine (PCP) use differed by Fiebig stage (*p* = .01), but there were few users of these drugs (opiates = 9, PCP = 18), compared to other drugs (ex., poppers = 56, marijuana = 92). Only about one third of participants reported having a weekly alcoholic drink in the past 4 months, and less than 10% reported any recent binge drinking (Table 3).

**Figure 1.**
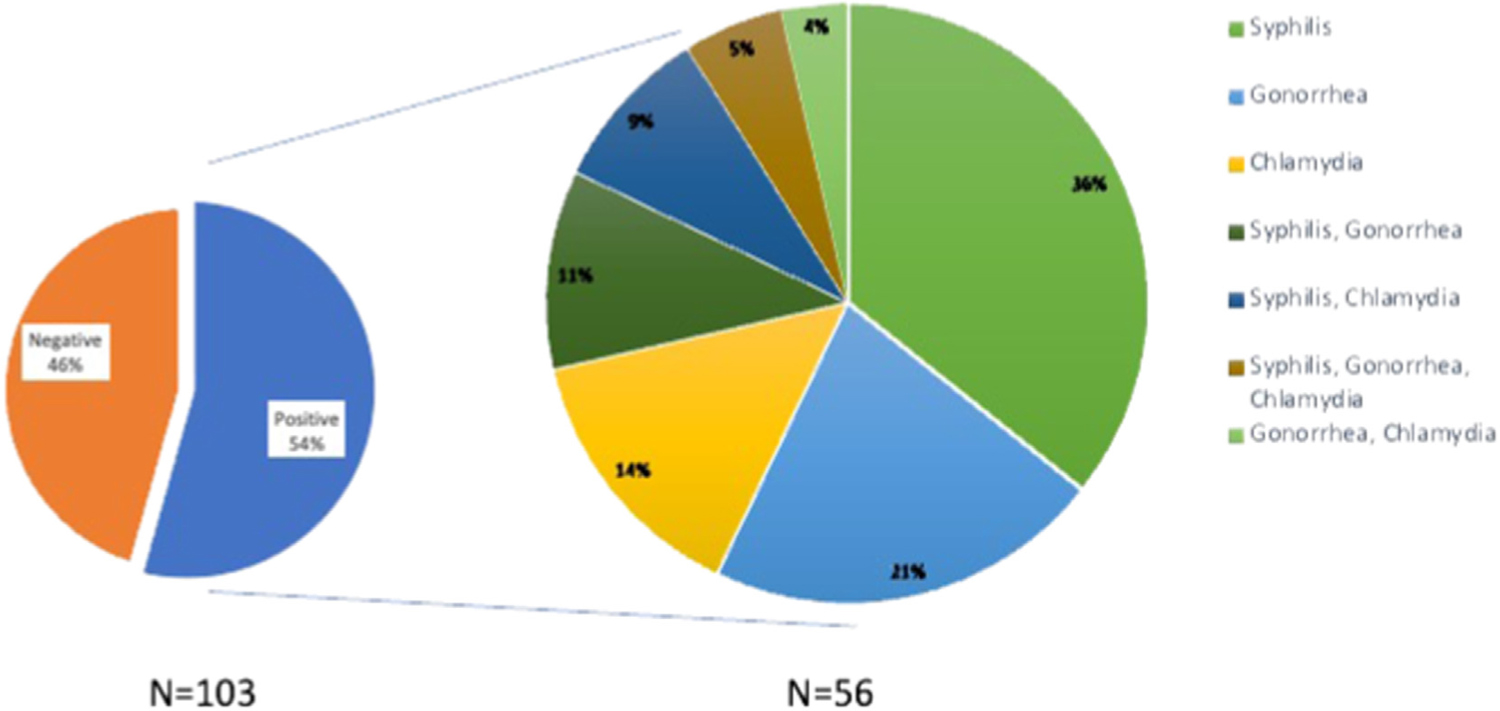
Sexually transmitted coinfections at baseline.

An STI coinfection of Chlamydia, Gonorrhea, and/or Syphilis was seen in 56 (54%) of participants (Figure 1). An STI coinfection did not differ by site or recruitment source nor correlated with VL, CD4 count, Fiebig stage, reported symptoms at enrollment, or relationship status (Table 4). Syphilis was the most frequent coinfection, with 33% (N = 34) testing positive at baseline, followed by Gonorrhea (N = 23, 22%), and Chlamydia (N = 18, 14%). Sixteen (17%) participants had more than one STI coinfection at baseline (Figure 1). When syphilis infection at baseline was examined, there were no significant differences in variables; however, there was a larger median VL in the participants with a syphilis coinfection (data not shown).

## Discussion

Adolescent Trials Network (ATN) 147 successfully enrolled youth at the time of HIV diagnosis followed by prompt ART initiation. While some participants were referred to the study from outside sources, most participants were either recruited after diagnosis of HIV-infection during screening for enrollment to ATN 149 or another study or were ATN 149 participants (30% of ATN 147 enrollees) who seroconverted during follow-up. Participants in the ATN 149 cohort were comprised of youth at a high risk for contracting HIV and were tested for HIV approximately every 4 months [19]. However, even with this high frequency of screening, almost half of the participants who contracted HIV while followed in the ATN 149 study were found to be Fiebig Stage VI, suggesting that even more frequent screening is necessary to capture acute infections. Additionally, acute retroviral symptoms were reported by less than half of the recruited youth, demonstrating that broad universal HIV screening is needed for better identification of recent infection in youth. However, while capturing acute HIV infection withing the first 3 months of infection is most likely to have the biggest effects of suppressing plasma VL and reducing HIV cellular viral reservoirs, individuals in this cohort were young and recently infected even if greater than 3 months. Intervening even after 3 months of infection may still greatly favor reduction in VL burden and preservation of normal immunity. Rapid and persistent suppression of viremia to levels below detection during the first year of infection can be highly beneficial by limit viral spread.

The demographics of our study population mirrored that of national health statistics for HIV-infected youth [17], once again highlighting health inequities pervasively prevalent in YLHIV. Transwomen comprised 4% of our population, which is twice the national incidence of HIV acquisition in transwomen [25], suggesting this may be an overlooked prevailing group in YLHIV. Additionally, few participants were IV drug users indicating that most HIV transmissions likely came from sexual contact. We did not observe associations between acute or established HIV infection status and demographic parameters, except that most acutely HIV-infected participants were enrolled in Los Angeles as compared to New Orleans. This most likely reflects slightly different recruitment practices at individual study sites although geographic variation in the timing of identification of HIV infection in youth could also play a role.

A high STI burden was present in most of the HIV-infected youth at the time of HIV diagnosis. Sexually transmitted infections (STIs), particularly syphilis, have been shown to facilitate HIV acquisition both sexually and perinatally, with a two to 5-fold HIV acquisition risk. [26–30]. In our cohort, no differences were seen in STI prevalence between the acutely infected youths, and those who had been infected for over 90 days. In the latter group, we do not have information linking syphilis infection to HIV infection, as we were not able to pinpoint the exact timing of both infections. For this reason, it is difficult to assess to what extent STIs facilitated HIV acquisition, given their high prevalence overall. At the time of HIV diagnosis, differences in HIV viral burden or absolute CD4 cell counts were not observed in patients with or without STIs, but it remains to be seen whether STIs could influence HIV viral kinetics over time. Drug use, which has been associated with STI acquisition [31] tended to be higher in acutely infected HIV participants, particularly PCP and opiates, but no significant differences were noted in other drug or alcohol use.

Thirty participants attempted suicide in their lifetime, and an additional six participants attempted suicide 4 months before enrollment which is much higher than the 1.9% national average of reported suicide attempts for the 18–25 age group [32]. Even with this high rate of mental health crisis in this population, only a quarter ever received outpatient services for mental health. Poor mental health has not only been associated with sexual risk behaviors [33] that could enhance acquisition risk, but is also detrimental to long term HIV health outcomes [34]. These statistics display a clear need for further integration of mental health services for young people at risk of and already living with HIV.

The median level of viremia of an acutely HIV-infected youth was 2.6 times higher than the median value of someone with nonacute infection. Higher levels of viremia associated with acute HIV infection have important implications for HIV transmission and disease prognosis [35–37]. The ability to recruit and initiate ART when viral burden is highest during early/acute phase in YLHIV likely results in more favorable HIV disease prognosis in youth who traditionally may have more pliable HIV reservoirs than adults due to an active thymus [38–40], as compared to youth who initiate treatment later after immune disruption [41]. Early ART after diagnosis has resulted in improved HIV control and outcomes in both adults [42] and in children under the age of 17 [43], however how early ART affects HIV progression in young adults (16–24) has not been thoroughly investigated. Our cohort also has a unique advantage of knowing Fiebig stage at the time of ART initiation, allowing for further investigation into how early detection may be important to improving HIV outcomes.

In summary, ATN 147 successfully recruited newly diagnosed YLHIV followed by prompt initiation of ART, with a high proportion of acutely HIV infections identified. Few demographic or behavioral differences were seen by infection duration; however, biological differences seen between the groups might suggest that early identification of HIV may contribute to improved disease outcomes [43]. Youth living with HIV (YLHIV) reflect the pervasive health disparities in our society, with the vast majority being of non-White race/ethnicity and often reporting experiences such as suicide attempts that may be reflective of social, economic and health inequities, and early detection could help prevent further spread and better outcomes. Acute retroviral syndrome was not identified in nearly 2/3 of participants which limits its usefulness in identifying novel infections. We hope that the early diagnoses and intervention of our participants will provide beneficial HIV outcomes.

## Figures and Tables

**Table 1:** Population demographics (N = 103)

	Median	IQR
RNA viral load (copies/ml)	37,313	(5,849–126,162)
CD4 count (N = 100)	445.5	(357–613)

Age	N	*%*
Average (sd)	20.8	2.3
16–18 years	21	20.4%
19–21 years	39	37.9%
22–24 years	43	41.7%
Coinfection STI at baseline		
Yes	56	54.3%
Race/Ethnicity		
Black or African-American	62	60.2%
Asian/Native American/Alaska Native/Pacific Islander	7	6.8%
Latino	26	25.2%
White	8	7.8%
Gender identifying		
Cis male	93	90.3%
Cis female	2	1.9%
Trans female	5	4.9%
Gender nonconforming	3	2.9%
Sexual orientation		
Gay/Same gender	74	71.8%
Heterosexual	5	4.9%
Bisexual	19	18.4%
Other	5	4.9%
Housing		
Recently homeless	11	10.7%
Ever homeless	25	24.3%
Relationship		
Monogamous	12	11.7%
Single or one of more casual relationships	86	83.5%
Other/Did not Answer	5	4.9%
Employment		
Student	30	29.1%
Full/Part/Self-employed	51	49.5%
Unemployed	18	17.5%
Did not answer	4	3.9%
Education		
Below high school	17	16.5%
High-school diploma/equivalent	26	25.2%
Some higher education	54	52.4%
Completed higher education	4	3.9%
Missing	2	1.9%
Site		
Los Angeles	51	49.5%
New Orleans	52	50.5%
Symptoms at enrollment		
Before	13	12.6%
At enrollment	28	27.2%
None reported	62	60.2%
Insurance		
Private	18	17.5%
MediCal, Medicare, ADAP or other assistance	77	74.8%
Other or unknown	8	7.8%
ART start date		
Within 24 hours of enrollment	70	68.0%
Within 48 hours of enrollment	10	9.7%
Within one week of enrollment	10	9.7%
More than one week/unknown	13	12.6%

ART = antiretroviral treatment; IQR = interquartile range; STI = sexually transmitted infection.

**Table 2:** Clinical and demographic variables stratified by Fiebig stage

	By Fiebig stage				
	Acute (Fiebig I-V)		Established (Fieabig VI)		*p* value
	N = 36		N = 67		
	Median	IQR	Median	IQR	
RNA Viral Load (copies/ml)^a^	86,900	9,742–559,230	32,334	3,755–76,552	**.006**
CD4 count cells/ml (N = 100)^a^	517	419–549	424	343–458	**.02**

Age	N	%	N	%	*p* value
Average (sd)^b^	21.1	2.42	20.6	2.15	.29
16–18 years	7	19.4%	14	20.9%	.70
19–21 years	12	33.3%	27	40.3%	
22–24 years	17	47.2%	26	38.9%	
Coinfection STI					
Yes	21	58.3%	35	52.2%	.55
Race/Ethnicity					
Black or African-American	17	47.2%	45	67.2%	.15
Asian/Native American/Alaska Native/Pacific Islander	4	11.1%	3	4.5%	
Latino	11	20.6%	15	22.4%	
White	4	11.1%	4	5.6%	
Gender Identifying					
Cis male	31	86.1%	62	92.5%	.64
Cis female	1	2.8%	1	1.5%	
Trans female	3	8.3%	2	3.0%	
Gender nonconforming	1	2.8%	2	3.0%	
Sexual orientation					
Gay/Same gender	26	72.2%	48	71.6%	.98
Heterosexual	2	5.6%	3	3.0%	
Bisexual	6	16.7%	13	19.4%	
Other	2	5.6%	3	3.0%	
Site					
Los Angeles	22	61.1%	29	42.19%	.08
New Orleans	14	38.9%	38	57.81%	
Recruitment Source					
outside referral	20	55.6%	52	77.6%	**.02**
Recruited from ATM 149	16	44.4%	15	22.4%	
Symptoms at enrollment					
Before	0	0.0%	13	19.4%	**.008**
At enrollment	14	38.9%	14	20.9%	
None reported	22	61.1%	40	59.7%	
Insurance^c^					
Private	8	22.2%	10	14.9%	.16
Medi-Cal, ADAP, or other assistance	28	77.8%	49	73.1%	
Other or unknown	0	0.0%	8	11.9%	

IQR = interquartile range; STI = sexually transmitted infection.

*p* values for categorical variables were calculated by X^2^, or Fisher’s test when appropriate.

Bolded values are below .05 significance level.

a *p* values for Viral Load and CD four count are calculated with the Wilcoxon Rank-Sum Test.

b *p* values for age were calculated with Student’s t-test.

c All participants were given insurance when enrolling (if they had none) through Medi-cal, Ryan White, or other assistance insurance.

**Table 3:** Behavioral factors stratified by Fiebig stage

	Fiebig stage				
	Acute (Fiebig I-V)		Established (Fiebig VI)		*p* value
	N = 36		N = 67		
	N	%	N	%	
Employment					
Student	10	27.8%	20	29.9%	.81
Full/Part/Self-employed	19	52.8%	32	47.8%	
Unemployed	5	13.8%	13	19.4%	
Did not answer	2	5.6%	2	3.0%	
Education					
Below high school	8	22.2%	9	13.4%	.41
High-school diploma/equivalent	10	27.8%	16	23.9%	
Some higher education	15	41.7%	39	58.2%	
Completed higher education	2	5.6%	2	3.0%	
Missing =2					
Housing					
Recent homeless	6	16.7%	5	7.5%	.29
Ever homeless	11	30.6%	14	20.9%	.62
Relationship					
Monogamous	3	8.3%	9	13.4%	.39
Single or one of more casual Relationships	30	83.3%	56	83.6%	
Other/Did not answer	3	8.3%	2	3.0%	
HIV positive partner within 4 months					
Yes	14	38.9%	22	32.8%	.69
No	9	25.0%	22	32.8%	
Unknown/Missing	13	36.1%	23	34.3%	
Sex exchange					
Recent	4	11.1%	5	7.5%	.79
Ever	9	25.0%	8	11.9%	.23
Mental health					
Ever Hospitalized for psychiatric condition	3	8.3%	2	3.0%	.34
Hospitalized for psychiatric condition in last 4 months	2	5.6%	0	0.0%	.16
Attempted suicide ever	15	41.7%	15	22.4%	.14
Attempted suicide in last 4 months	1	2.8%	5	7.5%	.41
Ever received outpatient services for mental heaLth	8	22.2%	17	25.4%	.66
Received outpatient services for mental health in last 4m	7	19.4%	11	16.4%	.66
Recent Alcohol/Drug use					
Alcohol at least once week in the last 4m	13	36.1%	19	28.4%	.16
5+ drinks at least once a week in the Last 4m	4	11.1%	5	7.5%	.77
Crack	6	16.7%	8	11.9%	.55
Ecstasy	1	2.8%	7	10.4%	.11
Heroine	1	2.8%	0	0.0%	.14
K2	1	2.8%	1	1.5%	.53
Ketamine	1	2.8%	0	0.0%	.14
Methamphetamines	3	8.3%	5	7.5%	.74
Opiates	3	8.3%	3	4.5%	.43
PCP	5	13.9%	3	4.5%	.21
Poppers	13	33.3%	18	28.4%	.33
Marijuana	22	61.1%	48	71.2%	.47
IV drug use	1	2.8%	0	0.0%	.26
Lifetime Drug Use	14	38.9%	18	26.9%	.16
Crack					
Ecstasy	13	36.1%	14	20.1%	.09
Heroine	1	2.8%	2	3.0%	.95
K2	3	8.3%	6	9.0%	.76
Ketamine	3	8.3%	1	1.5%	.14
Methamphetamines	6	16.7%	6	9.0%	.26
Opiates	6	16.7%	3	4.5%	**.04**
PCP	12	33.3%	6	9.0%	**.001**
Poppers	20	55.6%	26	38.8%	.49
Marijuana	32	88.9%	60	89.5%	.77
IV drug use	3	8.3%	1	1.5%	.08

PCP = phencyclidine.

**Table 4:** Potential Associations between enrollment parameters and sexually transmitted coinfections at baseline

	Presence of STI (N = 56)		No STI (N = 47)		*p* value
	Median	IQR	Median	IQR	
RNA Viral Load (Log10)	38,050	(9,741–129,101)	35,042	(3,587–113,000)	.35
CD4 count (n = 100)	459	(267–630)	427	(315–571)	.24

	N	%	N	%	*p* value
Fiebig Stage					
Acute	21	41.1%	15	34.0%	.55
Established	35	58.9%	32	66.0%	
Site					
Los Angeles	28	50.0%	23	48.9%	.19
New Orleans	28	50.0%	24	51.1%	
Relationship					
Monogamous	4	7.1%	8	17.0%	.17
Single or one of more casual relationships	48	85.7%	38	80.9%	
Other/Did not Answer	4	7.1%	1	2.1	
Symptoms at enrollment					
Before	7	12.5%	6	12.8%	.93
At enrollment	15	26.8%	13	27.7%	
None reported	34	60.7%	28	59.6%	

## References

[1] Stricof RL, Kennedy JT, Nattell TC, HIV seroprevalence in a facility for runaway and homeless adolescents. Am J Public Health 1991;81(Suppl): 50–3. 2014885 10.2105/ajph.81.suppl.50 PMC1404745

[2] Bekker LG, Johnson L, Wallace M, Hosek S. Building our youth for the future. J Int AIDS Soc 2015;18(2 Suppl 1):20027. 25724512 10.7448/IAS.18.2.20027 PMC4344540

[3] Nielsen-Saines K, Mitchell K, Kerin T, Acute HIV infection in youth: Protocol for the adolescent trials network 147 (ATN147) comprehensive adolescent research and engagement studies (CARES) study. JMIR Res Protoc 2019;8:e10807. 30650057 10.2196/10807 PMC6351983

[4] Cotton MF, Violari A, Otwombe K, Early time-limited antiretroviral therapy versus deferred therapy in South African infants infected with HIV: Results from the children with HIV early antiretroviral (CHER) randomised trial. Lancet 2013;382:1555–63. 24209829 10.1016/S0140-6736(13)61409-9 PMC4104982

[5] Shiau S, Abrams EJ, Arpadi SM, Kuhn L. Early antiretroviral therapy in HIV-infected infants: Can it lead to HIV remission? Lancet HIV 2018;5:e250e8. 29739699 10.1016/S2352-3018(18)30012-2 PMC7487171

[6] Shiau S, Strehlau R, Shen Y, Virologic response to very early HIV treatment in neonates. J Clin Med 2021;10:2074. 34066021 10.3390/jcm10102074 PMC8151270

[7] Violari A, Cotton MF, Gibb DM, Early antiretroviral therapy and mortality among HIV-infected infants. N Engl J Med 2008;359: 2233–44. 19020325 10.1056/NEJMoa0800971 PMC2950021

[8] Lodi S, Costagliola D, Sabin C, Effect of immediate initiation of antiretroviral treatment in HIV-positive individuals aged 50 years or older. J Acquir Immune Defic Syndr 2017;76:311–8. 28746165 10.1097/QAI.0000000000001498 PMC5704899

[9] Lodi S, Meyer L, Kelleher AD, Immunovirologic control 24 months after interruption of antiretroviral therapy initiated close to HIV seroconversion. Arch Intern Med 2012;172:1252–5. 22826124 10.1001/archinternmed.2012.2719

[10] Saag MS, Gandhi RT, Hoy JF, Antiretroviral drugs for treatment and prevention of HIV infection in adults: 2020 recommendations of the international antiviral society-USA panel. JAMA 2020;324:1651–69. 33052386 10.1001/jama.2020.17025 PMC11017368

[11] Severe P, Juste MA, Ambroise A, Early versus standard antiretroviral therapy for HIV-infected adults in Haiti. N Engl J Med 2010;363:257–65. 20647201 10.1056/NEJMoa0910370 PMC3676927

[12] Volberding P, Demeter L, Bosch RJ, Antiretroviral therapy in acute and recent HIV infection: A prospective multicenter stratified trial of intentionally interrupted treatment. AIDS 2009;23:1987–95. 19696651 10.1097/QAD.0b013e32832eb285 PMC2888600

[13] Sáez-Cirión A, Bacchus C, Hocqueloux L, Post-treatment HIV-1 controllers with a long-term virological remission after the interruption of early initiated antiretroviral therapy ANRS VISCONTI Study. PLoS Pathog 2013;9:e1003211. 23516360 10.1371/journal.ppat.1003211 PMC3597518

[14] Williams JP, Hurst J, Stöhr W, HIV-1 DNA predicts disease progression and post-treatment virological control. Elife 2014;3:e03821. 25217531 10.7554/eLife.03821 PMC4199415

[15] Krebs SJ, Ananworanich J. Immune activation during acute HIV infection and the impact of early antiretroviral therapy. Curr Opin HIV AIDS 2016; 11:163–72. 26599167 10.1097/COH.0000000000000228

[16] CDC CfDCaP. HIV surveillance - adolescents and young adults. 2017. Available at: https://www.cdc.gov/hiv/pdf/library/slidesets/cdc-hiv-surveillance-adolescents-young-adults-2017.pdf. Accessed October 1, 2022.

[17] CDC. Centers for Disease Control and Prevention. CDCHIV surveillance-adolescents and young adults 2018. 2018. Available at: https://www.cdc.gov/hiv/pdf/library/slidesets/cdc-hiv-surveillance-adolescents-young-adults-2018.pdf. Accessed October 1, 2022.

[18] Swendeman D, Arnold EM, Harris D, Text-messaging, online peer support group, and coaching Strategies to optimize the HIV prevention continuum for youth: Protocol for a randomized controlled trial. JMIR Res Protoc 2019;8:e11165. 31400109 10.2196/11165 PMC6707028

[19] Rotheram MJ, Fernandez MI, Lee SJ, Strategies to treat and prevent HIV in the United States for adolescents and young adults: Protocol for a mixed-methods study. JMIR Res Protoc 2019;8:e10759. 30664482 10.2196/10759 PMC6360384

[20] Fiebig EW, Wright DJ, Rawal BD, Dynamics of HIV viremia and antibody seroconversion in plasma donors: Implications for diagnosis and staging of primary HIV infection. AIDS 2003;17:1871–9. 12960819 10.1097/00002030-200309050-00005

[21] Arnold EM, Swendeman D, Harris D, The stepped care intervention to suppress viral load in youth living with HIV: Protocol for a randomized controlled trial. JMIR Res Protoc 2019;8:e10791. 30810536 10.2196/10791 PMC6414817

[22] Services. DoHaH. Panel on antiretroviral guidelines for adults and adolescents. Guidelines for the use of antiretroviral agents in adults and adolescents, with HIV. 2022. Available at: https://clinicalinfo.hiv.gov/sites/default/files/guidelines/documents/AdultandAdolescentGL.pdf. Accessed October 1, 2022.

[23] Comulada WS, Tang W, Swendeman D, Development of an electronic data collection system to support a large-scale HIV behavioral intervention trial: Protocol for an electronic data collection system. JMIR Res Protoc 2018;7:e10777. 30552083 10.2196/10777 PMC6315223

[24] R Core Team. R: A language and environment for statistical computing. Vienna, Austria: R Foundation for Statistical Computing; 2021. Available at: https://www.R-project.org/.Team. Accessed November 8, 2023.

[25] Prevention. CfDCa. Behavioral and clinical characteristics of persons with diagnosed HIV infectiondmedical monitoring project, United States, 2018 cycle (June 2018eMay 2019). Available at: https://www.cdc.gov/hiv/library/reports/hiv-surveillance.html. Accessed May 1, 2023.

[26] Buchacz K, Patel P, Taylor M, Syphilis increases HIV viral load and decreases CD4 cell counts in HIV-infected patients with new syphilis infections. AIDS 2004;18:2075–9. 15577629 10.1097/00002030-200410210-00012 PMC6763620

[27] Kalichman SC, Pellowski J, Turner C. Prevalence of sexually transmitted coinfections in people living with HIV/AIDS: Systematic review with implications for using HIV treatments for prevention. Sex Transm Infect 2011; 87:183–90. 21330572 10.1136/sti.2010.047514 PMC4317792

[28] Harrington P, Onwubiko U, Qi M, Factors associated with HIV seroconversion among women attending an urban health clinic in the south: A matched case-control study. AIDS Patient Care STDS 2020;34:124–31. 32109142 10.1089/apc.2019.0259

[29] Yeganeh N, Watts HD, Camarca M, Syphilis in HIV-infected mothers and infants: Results from the NICHD/HPTN 040 study. Pediatr Infect Dis J 2015;34:e52–7. 25742089 10.1097/INF.0000000000000578 PMC4352722

[30] Melo MG, Sprinz E, Gorbach PM, HIV-1 heterosexual transmission and association with sexually transmitted infections in the era of treatment as prevention. Int J Infect Dis 2019;87:128–34. 31404674 10.1016/j.ijid.2019.08.004 PMC6894479

[31] Feaster DJ, Parish CL, Gooden L, Substance use and STI acquisition: Secondary analysis from the AWARE study. Drug Alcohol Depend 2016; 169:171–9. 27837708 10.1016/j.drugalcdep.2016.10.027 PMC5140686

[32] Garnett MF, Curtin SC, Stone DM. Suicide mortality in the United States, 2000–2020. NCHS Data Brief No 433 2022:1–8. 35312475

[33] Koegler E, Kennedy CE. A scoping review of the associations between mental health and factors related to HIV acquisition and disease progression in conflict-affected populations. Confl Health 2018;12:20. 29881448 10.1186/s13031-018-0156-y PMC5984364

[34] Remien RH, Stirratt MJ, Nguyen N, Mental health and HIV/AIDS: The need for an integrated response. AIDS 2019;33:1411–20. 30950883 10.1097/QAD.0000000000002227 PMC6635049

[35] Pilcher CD, Joaki G, Hoffman IF, Amplified transmission of HIV-1: Comparison of HIV-1 concentrations in semen and blood during acute and chronic infection. AIDS 2007;21:1723–30. 17690570 10.1097/QAD.0b013e3281532c82 PMC2673564

[36] Pilcher CD, Tien HC, Eron JJ, Brief but efficient: Acute HIV infection and the sexual transmission of HIV. J Infect Dis 2004;189:1785–92. 15122514 10.1086/386333

[37] Wawer MJ, Gray RH, Sewankambo NK, Rates of HIV-1 transmission per coital act, by stage of HIV-1 infection, in Rakai, Uganda. J Infect Dis 2005;191:1403–9. 15809897 10.1086/429411

[38] Ananworanich J, Puthanakit T, Suntarattiwong P, Reduced markers of HIV persistence and restricted HIV-specific immune responses after early antiretroviral therapy in children. AIDS 2014;28:1015–20. 24384692 10.1097/QAD.0000000000000178

[39] Hakim FT, Cepeda R, Kaimei S, Constraints on CD4 recovery post-chemotherapy in adults: Thymic insufficiency and apoptotic decline of expanded peripheral CD4 cells. Blood 1997;90:3789–98. 9345067

[40] Mackall CL, Fleisher TA, Brown MR, Age, thymopoiesis, and CD4 T-lymphocyte regeneration after intensive chemotherapy. N Engl J Med 1995;332:143–9. 7800006 10.1056/NEJM199501193320303

[41] Bitnun A, Ransy DG, Brophy J, Clinical correlates of human immunodeficiency virus-1 (HIV-1) DNA and inducible HIV-1 RNA reservoirs in peripheral blood in children with perinatally acquired HIV-1 infection with sustained virologic suppression for at least 5 years. Clin Infect Dis 2020;70: 859–66. 30919879 10.1093/cid/ciz251 PMC7319270

[42] Lundgren JD, Babiker AG, Gordin F, Initiation of antiretroviral therapy in early asymptomatic HIV infection. N Engl J Med 2015;373:795–807. 26192873 10.1056/NEJMoa1506816 PMC4569751

[43] Frange P, Montange T, Le Chenadec J, Impact of early versus late antiretroviral treatment initiation on naive T lymphocytes in HIV-1-Infected children and adolescents - the-ANRS-EP59-CLEAC study. Front Immunol 2021;12:662894. 33968064 10.3389/fimmu.2021.662894 PMC8100053

